# High steam-conditioning temperature during the pelleting process impairs growth performance and nutrient utilization in broiler starters fed barley-based diets, regardless of carbohydrase supplementation

**DOI:** 10.1016/j.psj.2021.101166

**Published:** 2021-03-27

**Authors:** W.N.U. Perera, M.R. Abdollahi, F. Zaefarian, T.J. Wester, V. Ravindran

**Affiliations:** ⁎ Monogastric Research Centre, School of Agriculture and Environment, Massey University, Palmerston North 4442, New Zealand; † Department of Animal Science, Faculty of Agriculture, University of Peradeniya, Peradeniya 20400, Sri Lanka

**Keywords:** barley, broiler, carbohydrase, conditioning temperature, enzyme

## Abstract

The influence of supplemental carbohydrase (**Carb**) and conditioning temperature (**CT**) on growth performance, nutrient utilization and intestinal morphometry of broilers (d 1–21) fed barley-based diets was examined in a 2 × 3 factorial arrangement, evaluating 2 levels of Carb (0 and 150 g/tonne of feed) and three CT (60, 74, and 88°C). A total of 288, 1-day-old male broilers (8 birds/cage; 6 cages/treatment) were used. The activities of endo-1,4-β- glucanase, endo-1,3 (4)-β-glucanase and endo-1,4-β-xylanase in the Carb were 800 BGU/g, 700 BGU/g and 2,700 XU/g, respectively. On d 21, ileal digesta was collected for the determination of nutrient digestibility. There was no significant interaction between Carb and CT for any tested parameter. Supplemental Carb, regardless of CT, increased weight gain (**WG**; *P* < 0.05) and reduced feed per gain (**F/G**; *P* < 0.001) by 30 g/bird and 6.5 points, respectively. Increasing CT to 88°C reduced (*P* < 0.05) WG, but increased (*P* < 0.05) F/G compared to the diets conditioned at 60° and 74°C. Regardless of CT, Carb enhanced (*P* < 0.05) the digestibility of starch and AMEn by 1.15% and 32 kcal/kg, respectively. Compared to the diets conditioned at 60° and 74°C, CT at 88°C reduced (*P* < 0.05) digestibility of dry matter, nitrogen, phosphorus, gross energy, and AMEn. Birds fed diets conditioned at 88°C showed lower (*P* < 0.05) starch digestibility compared to those fed diets conditioned at 60°C. Conditioning at 88°C increased (*P* < 0.05) jejunal digesta viscosity by 10.2% compared to diets conditioned at 60° and 74°C. Overall, Carb supplementation improved WG, F/G, starch digestibility and AMEn in broilers fed barley-based diets, irrespective of CT applied. Conditioning barley-based diets at 88°C impaired the ability of birds to utilize nitrogen, starch, phosphorus and energy, and consequently deteriorated WG and F/G. The lack of significant interactions between Carb and CT indicated that negative impacts caused by high CT on bird performance and nutrient utilization occurred regardless of Carb enzyme supplementation. Supplemental Carb *per se* could not remedy the adverse effects of high CT.

## INTRODUCTION

The use of barley in poultry diets is limited due mainly to its high content of soluble non-starch polysaccharides (**NSP**) that results in increased intestinal digesta viscosity, leading to impaired nutrient utilization and performance of birds fed barley-based diets. Different heat processing methods such as steam-cooking (Gracia et al., 2003), expansion (García et al., 2008), extrusion (Vranjes and Wenk, 1995) and micronization (Zheng et al., 1998; García et al., 2008) have been evaluated as potential methods to enhance the feeding value of barley in poultry diets. Expansion, extrusion and micronization are short-time high-temperature processes that involve temperature >100°C. Heat processing is believed to disrupt the cell structures and to release the encapsulated nutrients (Gracia et al., 2003; García et al., 2008) facilitating the nutrient utilization. However, thermal processing can increase solubilization of NSP (Silversides and Bedford, 1999), leading to greater viscosity in both feed and intestinal contents particularly in diets based on viscous grains such as barley (Svihus et al., 2000; Cowieson et al., 2005; García et al., 2008). Accordingly, to achieve the desired outcome of the thermal processing, the application of optimum conditions during feed manufacture is vital.

During the pelleting process, high conditioning temperatures (**CT**; >80°C) are commonly employed by poultry feed manufacturers to obtain high-quality pellets (Cutlip et al., 2008; Abdollahi et al., 2013) and to maintain feed hygiene by controlling foodborne pathogens, such as *Salmonella* and *Campylobacter* (Amerah et al., 2011; Abdollahi et al., 2013). High CT, however, can result in the formation of resistant starch (Abdollahi et al., 2011; Abdollahi et al., 2010b), degradation of heat-labile amino acids (Papadopoulos, 1989), inactivation of vitamins (Jensen, 2000) and supplemental enzymes (Inborr and Bedford, 1994), reduced nutrient utilization (Abdollahi et al., 2010a,b) and compromised growth performance (Cutlip et al., 2008; Abdollahi et al., 2011). Impaired nutrient utilization of birds fed diets conditioned at higher CT can be attributed to losses of different nutrients in feed (Papadopoulos, 1989) and viscosity-induced interference of nutrient absorption (Smulikowska et al., 2002) in the gastrointestinal tract.

On the other hand, lower CT can hinder the inactivation of antinutritive factors and result in insufficient starch gelatinization, protein denaturation and poor pellet quality (Abdollahi et al., 2013; Netto et al., 2019), while failing to assure satisfactory feed hygiene (Boney et al., 2018). Effects of CT also vary depending on the grain type (Abdollahi et al., 2010a,b). All of these illustrate the importance of determining the optimum CT of feed for each grain type used. The influence of CT on growth performance and nutrient utilization of broilers fed corn- (Cutlip et al., 2008; Abdollahi et al., 2010a,b; Loar II et al., 2014), wheat- (Abdollahi et al., 2010a, 2011) and sorghum- (Abdollahi et al., 2010b) based diets are better understood. Whilst the optimum inclusion level (Perera et al., 2019b) and particle size (Perera et al., 2020b) for barley in pelleted broiler diets have been evaluated, the optimum CT for pelleting barley-based diets remains unexplored.

The NSP-degrading enzymes are routinely added to barley-based diets to overcome the adverse effects of anti-nutritional factors, mainly the greater digesta viscosity. Improved performance and nutrient utilization from supplemental enzymes in birds fed barley-based diets are attributed mostly to the reduction of digesta viscosity (Almirall et al., 1995; Perera et al., 2019b). As high CT may exacerbate the adverse effects of viscosity in diets based on viscous grains such as barley, use of exogenous enzymes becomes even more critical (Cowieson et al., 2005). A better understanding of possible interactions between NSP-degrading enzyme and CT, particularly on intestinal digesta viscosity, and whether enzymes are more effective in barley-based diets conditioned at higher CT, would allow the poultry industry to optimize the use and potential of barley in poultry diets. Accordingly, the objectives of this study were set to evaluate whether interactive effects between supplemental carbohydrase (**Carb**) and CT exist on the performance, energy and nutrient utilization, and gut morphometry in broiler starters fed barley-based diets.

## MATERIALS AND METHODS

### Enzymes

A multicomponent NSP-degrading enzyme, Ronozyme Multigrain, (produced by *Trichoderma reesei,* also known as *Trichoderma longiabrachiatum*) and Ronozyme HiPhos were obtained from DSM Nutritional Products, East Wagga Wagga, Australia. The activities of endo-1,4-β-glucanase, endo-1,3(4)-β-glucanase and endo-1,4-β-xylanase in Ronozyme Multigrain were 800 BGU/g, 700 BGU/g and 2,700 XU/g, respectively. One unit of β-glucanase (**BGU**) is defined as the quantity of enzyme that releases 1.0 µmol of reducing moieties from 1.5% β-glucan per minute at pH 5.0 at incubation temperature of 40°C for 20 min. One unit of xylanase (**XU**) is defined as the quantity of enzyme that releases 1.0 µmol of reducing moieties from 1.5% arabinoxylan per minute at pH 5.0 and incubation temperature of 40°C for 20 min. Ronozyme HiPhos was a granular 6-phytase preparation expressed by submerged fermentation of *Aspergillus oryzae* and contained > 10,000 phytase units (**FYT**)/g. One FYT is defined as the activity of enzyme that releases 1.0 μmol of inorganic phosphorus (**P**)/min from 5.0 mM sodium phytate at pH 5.5 at 37°C (DSM Nutritional Products Ltd., 2013). The activities of phytase, endo-1,4-β-xylanase, endo-1,3 (4)-β-glucanase and endo-1,4-β-glucanase in the pelleted diet samples were measured at Biopract GmbH, Berlin, Germany. The enzyme recovery was calculated as the percentage of measured enzyme activity in the diet to the expected enzyme activity estimated from the amount and minimum activity (DSM Nutritional Products Ltd., 2013) of enzymes added to the diets.

### Diets

Normal-starch hulled barley (cultivar, Fortitude) was obtained from a seed multiplication company (Luisetti Seeds Ltd., Rangiora, New Zealand) and ground in a hammer mill to pass through the screen size of 8.0 mm. Nutrient composition, nitrogen-corrected apparent metabolizable energy (AMEn) and standardized digestible amino acid contents of normal-starch hulled barley determined in a previous study (Perera et al., 2019a), were used in formulating a basal diet to meet the Ross 308 strain recommendations for major nutrients for broiler starters (Ross, 2019; Table 1). Ronozyme HiPhos (DSM Nutritional Products, Australia) was used in the basal diet and phytase matrix values (1.5 g/kg non-phytate P and 1.8 g/kg calcium) were used in basal diet formulation. The basal diet was then used to develop two feed batches, without and with an NSP-degrading enzyme (Ronozyme Multigrain; DSM Nutritional Products, Australia). Each diet, without and with Carb, was divided into 3 equal batches and conditioned at 3 different temperatures (60°, 74°, and 88°C) by adjusting the steam flow rate. Mash diets were steam-conditioned for 30 s and the CT was measured at the outlet (close to the exit point) of the conditioner before the mash feed entered the pellet die. The CT of the mash was measured continuously as a single-point measure during the conditioning time. Following conditioning, all diets were pelleted using a pellet mill (Model Orbit 15; Richard Sizer, Kingston-upon-Hull, UK) capable of manufacturing 180 kg of feed/h and equipped with a die ring with 3.0 mm holes and 35 mm thickness. Diet samples after conditioning and before passing through the pellet die (conditioned-only), and the conditioned and pelleted samples were collected for the determination of gelatinized starch (**GS**) content. Another set of samples was collected after pelleting for the determination of pellet durability.

**Table 1:** Composition, calculated analysis and analyzed values (g/kg, as fed) of the basal diet.

*Item*		*Calculated analysis*	
Normal starch hulled barley	565.4	AMEn, kcal/kg	2,850
Soybean meal	316.1	Crude protein	238
Corn gluten meal	50.0	Digestible protein	196
Soybean oil	29.4	Digestible methionine	5.8
Di-calcium phosphate	11.5	Digestible methionine + cysteine	9.0
Limestone	8.2	Digestible lysine	12.2
L-Lysine HCl	3.1	Digestible threonine	8.2
DL-Methionine	2.4	Digestible arginine	13.1
L-Threonine	1.2	Digestible valine	9.5
Sodium chloride	1.8	Crude fat	42.1
Sodium bicarbonate	3.8	Crude fiber	44.7
Vitamin premix^1^	1.0	Calcium	9.6
Mineral premix^1^	1.0	Non-phytate phosphorus	4.8
Titanium dioxide^2^	5.0	Sodium	2.0
Pellet binder^3^	2.0	Chloride	2.0
Phytase^4^	0.1	Potassium	8.4
		*Analyzed values*	
		DM	916
		Gross energy, kcal/kg	4,084
		CP (Nitrogen × 6.25)	250
		Starch	315
		Fat	53.0
		Insoluble NSP^5^	136
		Soluble NSP	30.0
		Total NSP^6^	166
		GS^7^, g per 100 g total starch (conditioned-only)	
		60°C	11.4
		74°C	10.8
		88°C	13.2
		GS, g per 100 g total starch (conditioned-pelleted)	
		60°C	16.0
		74°C	15.4
		88°C	16.3

1 Supplied per kg of diet: antioxidant, 125 mg; biotin, 0.2 mg; calcium pantothenate, 20 mg; cholecalciferol, 5,000 IU; cyanocobalamin, 0.02 mg; folic acid, 2.0 mg; menadione, 4 mg; niacin, 80 mg; pyridoxine, 5.0 mg; trans-retinol, 15,000 IU; riboflavin, 9.0 mg; thiamine, 4.0 mg; dl-α-tocopheryl acetate, 80 IU; choline, 0.45 mg; ascorbic acid, 100 mg; Co, 1.0 mg; Cu, 20 mg; Fe, 40 mg; I, 2.0 mg; Mn, 100 mg; Mo, 1.0 mg; Se, 0.15 mg; Zn, 100 mg. Image Holdings Ltd., Auckland, New Zealand.

2 Merck KGaA, Darmstadt, Germany.

3 KEMBIND® (Kemin Industries [Asia] Pte Ltd) pellet binder, which contained modified lignosulphonate, guar gum, edible fatty acids and mineral oil, was added on top of each diet.

4 Ronozyme HiPhos (1,000 phytase units [FYT]/kg diet). One FYT is defined as the activity of enzyme that releases 1.0 μmole of inorganic phosphorus/min from 5.0 mM sodium phytate at pH 5.5 at 37 °C. Nutrient matrix values (0.15% non-Phytate P and 0.18% Ca) were used in basal diet formulation.

5 NSP, non-starch polysaccharides.

6 Total NSP= Insoluble NSP + Soluble NSP.

7 GS, gelatinized starch. Unconditioned diet contained 9.93 g gelatinized starch per 100 g total starch. Each value represents mean of 4 replicate samples. Non-supplemented diets (0 g/kg of Ronozyme Multigrain) were used in the analysis.

### Pellet Durability

Pellet durability of diets was determined in a Holmen Pellet Tester (New Holmen NHP100 Portable Pellet Durability Tester, TekPro Ltd., Willow Park, North Walsham, Norfolk, UK) using the method described by Abdollahi et al. (2010a). Briefly, clean pellet samples (100 g; 5 replicates per diet), with no fines, were rapidly circulated in an air stream around a perforated test chamber for 30 s. Resulting fines were removed continuously through perforations during the test cycle. After the test cycle, the pellets were ejected and weighed manually. The pellet durability index (**PDI**) was calculated as the percentage of weight of pellets not passing through the perforations at the end of the test to weight of whole pellets at the start.

### Birds and Housing

The experimental procedures were approved by the Massey University Animal Ethics Committee and complied with the New Zealand Code of Practice for the Care and Use of Animals for Scientific Purposes. A total of 288, 1-day-old male broilers (Ross 308), obtained from a commercial hatchery, were individually weighed and allocated to 36 cages in electrically heated battery brooders so that the average bird weight per cage was similar. Each of the 6 dietary treatments was randomly assigned to 6 cages, each housing 8 birds. The birds were transferred to grower cages on d 12 and continued on the same starter diets until the end of the trial (d 21). The space allocation per bird in brooder and grower cages was 530 and 640 cm^2^, respectively. The battery brooders and grower cages were housed in an environmentally controlled room with 20 h of fluorescent illumination per day. The temperature was maintained at 31°C on d 1 and was gradually reduced to 22°C by 21 d of age. The diets were offered ad libitum and water was available at all times.

### Performance Data

Body weights and feed intake (**FI**) were recorded on a cage basis at weekly intervals. Mortality was recorded daily. Feed per gain (**F/G**) values were corrected for the BW of any bird that died during the course of the experiment.

### Nitrogen-corrected Apparent Metabolizable Energy (AME_n_)

The AME_n_ was determined using the classical total excreta collection method. FI and total excreta output of each cage were quantitatively measured from d 17 to 20 post-hatch. Daily collections from each cage were pooled, mixed in a blender and subsampled. Subsamples were lyophilized (Model 0610, Cuddon Engineering, Blenheim, New Zealand), ground to pass through a 0.5 mm sieve and stored in airtight plastic containers at 4°C pending analysis. The diets and excreta samples were analyzed for DM, gross energy (**GE**), and nitrogen (**N**).

### Coefficient of Apparent Ileal Digestibility of Nutrients

On d 21, 6 broilers per cage were euthanized by intravenous injection (0.5 mL per kg live weight) of sodium pentobarbitone (Provet NZ Pty Ltd., Auckland, New Zealand), and digesta were collected from the lower half of the ileum by gently flushing with distilled water, as described by Ravindran et al. (2005). The ileum was defined as that portion of the small intestine extending from Meckel's diverticulum to a point ∼40 mm proximal to the ileocecal junction. The ileum was then divided into 2 halves and the digesta was collected from the lower half towards the ileocecal junction. Digesta from birds were pooled within a cage, frozen immediately after collection and subsequently lyophilized. The diets and lyophilized digesta samples were ground to pass through a 0.5-mm sieve and stored at 4°C until laboratory analysis. The diets and digesta samples were analyzed for DM, titanium (**Ti**), N, starch, fat, calcium, P, and GE.

### Gizzard pH and Jejunal Digesta Viscosity

In 2 birds from each replicate cage euthanized for ileal collection, gizzard pH was measured using a pH meter (pH spear, Oakton Instruments, Vernon Hill, IL). The glass probe was inserted directly through an opening made in the gizzard and placed in the digesta. Three values were taken from the proximal, middle and distal sections of gizzard and the average value was considered as the final pH value. The viscosity of jejunal digesta from 2 birds euthanized for ileal collection from each replicate cage was also measured. Digesta obtained from the lower jejunum was centrifuged at 3000 × *g* at 20°C for 15 min. A 0.5 mL aliquot of the supernatant was used in a viscometer (Brookfield digital viscometer, Model DV2TLV; Brookfield Engineering Laboratories Inc., Stoughton, MA) fitted with CP-40 cone spindle with shear rates of 5 to 500/s to measure the viscosity.

### Relative Length and Weight of Digestive Tract Segments

Two additional birds, with BW closest to the mean weight of the cage, were weighed and euthanized by cervical dislocation. The digestive tract from the crop to ceca was carefully excised and adherent fat was removed. The lengths of duodenum (pancreatic loop), jejunum (from the pancreatic loop to Meckel's diverticulum), ileum (from Meckel's diverticulum to ileo-cecal junction) and ceca were recorded as described by Amerah et al. (2008). The empty weights of crop, proventriculus, gizzard, duodenum, jejunum, ileum and ceca in individual birds were determined and reported as g/kg of BW.

### Chemical Analysis

Dry matter was determined using standard procedures (Method 930.15; AOAC, 2016). Nitrogen was determined by combustion (Method 968.06; AOAC, 2016) using a CNS-200 carbon, N and sulfur autoanalyzer (LECO Corporation, St. Joseph, MI). An adiabatic bomb calorimeter (Gallenkamp Autobomb, London, UK) standardized with benzoic acid was used for the determination of GE. Starch was measured using a Megazyme kit (Method 996.11; AOAC, 2016) based on thermostable α-amylase and amyloglucosidase (McCleary et al., 1997). Fat was determined using Soxtec extraction procedure for animal feed, forage and cereal grains (Method 2003.06; AOAC, 2016). Calcium and P were determined by colorimetric methods after ashing the samples at 550°C and acid digestion using 6.0 M HCl using standard procedures (method 968.08D; AOAC, 2016). Total, soluble and insoluble NSP were determined using an assay kit (Megazyme International Ireland Ltd., Wicklow, Ireland) based on thermostable α-amylase, protease and amyloglucosidase (Englyst et al., 1994). Gelatinized starch content of diet samples was determined using an assay kit (Megazyme International Ireland Ltd., Wicklow, Ireland). Samples were assayed for Ti on a UV spectrophotometer following the method of Short et al. (1996).

### Calculations

The AME of diets was calculated using the following formula:

$$\text{AM}E_{\text{diet}}\left(\text{kcal}/\text{kg}\right)=\left[\left(\text{FI}\times GE_{\text{diet}}\right)-\left(\text{Excreta}\;\text{output}\times GE_{\text{excreta}}\right)\right]/\text{FI}$$

Correction for zero N retention was made using a factor of 8.73 kcal per g N retained in the body (Hill and Anderson, 1958).

$$\text{AME}n_{\text{diet}}\left(\text{kcal}/\text{kg}\right)=\text{AM}E_{\text{diet}}-\left(8.73\times N\text{retention}\right)\;/\;1000$$

The coefficient of apparent ileal digestibility **(CAID)** of nutrients were calculated from the dietary ratio of nutrients to Ti relative to the corresponding ratio in the ileal digesta.

$$\text{CAID}\;\text{of}\;\text{nutrient}=\left[{\left(\text{Nutrient}\;/\;\text{Ti}\right)}_{d}-{\left(\text{Nutrient}\;/\;\text{Ti}\right)}_{i}\right]/{\left(\text{Nutrient}\;/\;\text{Ti}\right)}_{d}$$

where, (Nutrient / Ti)_d_ = ratio of nutrient to Ti in diet and (Nutrient / Ti)_i_ = ratio of nutrient to Ti in ileal digesta.

Ileal digestible energy **(IDE)** was calculated using the following formula.

$$\text{IDE}\left(\text{kcal}/\text{kg}\right)=GE_{\text{diet}}\times \text{CAID}\;\text{of}\;\text{GE}$$

### Statistical Analysis

The data were analyzed as a 2 × 3 factorial arrangement of treatments evaluating 2 levels of Carb supplementation and three CT. Cage served as the experimental unit. The general linear model procedure of SAS (version 9.4; SAS Institute Inc., Cary, NC) in a completely randomised design was used. Significant differences between means were separated by least significant difference test. Differences were deemed significant at *P* < 0.05.

## RESULTS

### Pellet Durability and Enzyme Recovery

As shown in Table 2, PDI improved (*P* < 0.05) with increasing CT, with a greater PDI for the diet conditioned at 88°C than the diet conditioned at 60°C (66.4 vs. 62.2%).

**Table 2:** Influence of carbohydrase (Carb) enzyme addition and conditioning temperature on weight gain (WG; g/bird), feed intake (FI; g/bird) and feed per gain (F/G; g feed/g gain) of broiler starters^1^ (0–21 d), and pellet durability index (PDI; %).

Enzyme addition	Conditioning temperature, (°C)	WG	FI	F/G	PDI^2^
No enzyme	60	1,040	1,405	1.365	-
	74	1,026	1,376	1.355	-
	88	938	1,360	1.452	-
Carb	60	1,064	1,369	1.288	-
	74	1,033	1,371	1.327	-
	88	996	1,357	1.363	-
SEM^3^		13.6	11.2	0.0217	-
Main effects					
Enzyme addition					
No enzyme		1,001^b^	1,380	1.391^a^	-
Carb		1,031^a^	1,366	1.326^b^	-
Conditioning temperature, (°C)					
	60	1,052^a^	1,387	1.327^b^	62.2^b^
	74	1,029^a^	1,373	1.341^b^	64.8^a^^,^^b^
	88	967^b^	1,358	1.408^a^	66.4^a^
Probabilities, *P* ≤					
Enzyme addition		0.011	0.122	0.001	-
Conditioning temperature		0.001	0.054	0.002	0.021
Enzyme addition × Conditioning temperature		0.175	0.272	0.355	-

a,b Means in a column not sharing common letters are different (*P* < 0.05).

1 Each value represents the mean of 6 replicates (8 birds per replicate).

2 Each value represents the mean of 5 replicate samples.

3 Pooled standard error of mean.

The recovery of phytase in pelleted diets at 60°, 74°, and 88°C was 153, 128, and 48.5%, respectively. The recovery of endo-1,4-β-xylanase was 81, 55 and 16% at 60°, 74°, and 88°C, respectively. The endo-1,4-β-glucanase recovery at 60°, 74°, and 88°C was 70, 50, and 0%, respectively. Moreover, endo-1,3 (4)-β-glucanase recovery at 60°, 74°, and 88°C was 62, 46, and 0%, respectively.

### Growth Performance

Mortality during the experiment was insignificant. Only 3 out of the 288 birds died, and the deaths were not related to any specific treatment. As summarized in Table 2**,** there was no interaction between Carb and CT for any of growth performance parameters. Addition of Carb, regardless of CT, increased weight gain (**WG**; *P* < 0.05) and reduced F/G (*P* < 0.001) by 30 g/bird and 6.5 points, respectively. Regardless of the Carb addition, WG (*P* < 0.001) and F/G (*P* < 0.01) was influenced by CT. Birds fed diets conditioned at 60° and 74°C had a similar (*P* > 0.05) WG, but were greater (*P* < 0.05) than those fed the diets conditioned at 88°C. Conditioning at 88°C increased (*P* < 0.05) F/G compared to the diets conditioned at 60° and 74°C. Birds fed diets conditioned at 88°C tended (*P* = 0.054) to have a lower FI than those fed diets conditioned at 60°C.

### Nutrient and Energy Utilization

As shown in Table 3, no interaction between supplemental Carb and CT was observed for CAID of any analyzed nutrient, IDE and AMEn. Supplemental Carb enhanced (*P* < 0.01) the starch digestibility. Birds offered diets conditioned at 88°C had lower (*P* < 0.05) digestibility of DM, N, P and GE compared to the birds fed diets conditioned at 60° and 74°C. Diets conditioned at 88°C also resulted in lower (*P* < 0.05) starch digestibility than diets conditioned at 60°C. Regardless of CT, supplemental Carb increased AMEn by 32 kcal/kg. Steam-conditioning at 88°C reduced (*P* < 0.05) both IDE and AMEn compared to the diets conditioned 60° and 74°C.

**Table 3:** Influence of carbohydrase (Carb) enzyme addition and conditioning temperature on coefficient of apparent ileal digestibility (CAID)^1^ of DM, nitrogen (N), fat, starch, calcium (Ca), phosphorus (P), gross energy (GE), ileal digestible energy (IDE; kcal/kg DM)^1^ and AMEn (kcal/kg DM)^2^ of 21-day-old broilers.

Enzyme addition	Conditioning temperature, (°C)	CAID							IDE	AMEn
		DM	N	Fat	Starch	Ca	P	GE		
No enzyme	60	0.648	0.792	0.936	0.963	0.475	0.618	0.660	2,941	2,979
	74	0.672	0.821	0.951	0.956	0.466	0.606	0.687	3,062	2,977
	88	0.617	0.752	0.921	0.951	0.458	0.512	0.633	2,818	2,934
Carb	60	0.654	0.818	0.925	0.973	0.453	0.606	0.670	2,985	3,025
	74	0.656	0.809	0.931	0.971	0.440	0.574	0.673	3,001	3,000
	88	0.597	0.772	0.891	0.958	0.423	0.479	0.614	2,735	2,958
SEM^3^		0.0139	0.0136	0.0193	0.0046	0.0272	0.0175	0.0136	60.6	15.8
Main effects										
Enzyme addition										
		0.646	0.788	0.936	0.956^b^	0.466	0.579	0.660	2,940	2,963^b^
		0.636	0.800	0.916	0.967^a^	0.439	0.553	0.652	2,907	2,995^a^
Conditioning temperature, (°C)										
	60	0.651^a^	0.805^a^	0.931	0.968^a^	0.464	0.612^a^	0.665^a^	2,963^a^	3,002^a^
	74	0.664^a^	0.815^a^	0.941	0.963^a^^,^^b^	0.453	0.590^a^	0.680^a^	3,031^a^	2,989^a^
	88	0.607^b^	0.762^b^	0.906	0.954^b^	0.440	0.495^b^	0.623^b^	2,777^b^	2,946^b^
Probabilities, *P* ≤										
Enzyme addition		0.381	0.310	0.211	0.007	0.226	0.079	0.496	0.502	0.021
Conditioning temperature		0.001	0.001	0.192	0.021	0.688	0.001	0.001	0.001	0.003
Enzyme × Conditioning temperature		0.591	0.347	0.884	0.705	0.974	0.802	0.536	0.541	0.714

a,b Means in a column not sharing common letters are different (*P* < 0.05).

1 Each value represents the mean of 6 replicates (6 birds per replicate).

2 Each value represents the mean of 6 replicates (8 birds per replicate) measured from d 17 to 20.

3 Pooled standard error of mean.

### Relative Length and Weight of Digestive Tract Segments, Gizzard pH, and Jejunal Digesta Viscosity

No interaction was observed between supplemental Carb and CT on the relative empty weight or length of any measured intestinal segment (Table 4). Supplemental Carb reduced (*P* < 0.05) the relative length of the ileum and small intestine, and tended to reduce the relative length of the duodenum (*P* = 0.076) and jejunum (*P* = 0.087). Increasing CT to 88°C tended (*P* = 0.093) to increase the relative weight of the gizzard. Birds offered diets conditioned at 88°C had lighter (*P* = 0.050) ceca weight compared to those fed diets conditioned at 60°C, and longer (*P* < 0.05) duodenum and jejunum compared to those fed diets conditioned at 60° and 74°C.

**Table 4:** Influence of carbohydrase enzyme addition and conditioning temperature on relative weight (g/kg of BW) of crop, proventriculus (Prov.), gizzard (Giz.), duodenum (Duo.), jejunum (Jej.), ileum (Ile.) and ceca, and relative lengths (cm/kg of BW) of Duo., Jej., Ile. and ceca, pH of the gizzard and jejunal digesta viscosity (cP) of 21-day-old broilers^1^.

Enzyme addition	Conditioning temperature, (°C)	Relative empty weight								Relative length					Giz. pH	Jej. digesta viscosity
		Crop	Prov.	Giz.	Duo.	Jej.	Ile.	Ceca	SI^3^	Duo.	Jej.	Ile.	Ceca	SI^3^		
No enzyme	60	2.38	3.55	11.7	4.93	11.2	7.09	2.46	23.2	20.6	55.2	61.3	24.8	137	2.35	3.06
	74	2.20	3.75	11.8	4.49	9.2	6.30	2.44	20.0	19.7	52.2	57.7	24.0	130	2.56	3.20
	88	2.65	3.65	12.1	5.07	10.5	6.64	2.11	22.2	22.1	57.3	61.0	24.8	140	2.91	3.55
Carb	60	2.31	3.40	11.5	4.92	10.2	6.62	2.60	21.7	19.3	49.5	54.6	24.3	123	2.60	3.20
	74	2.43	3.79	11.8	4.98	10.3	6.25	2.39	21.5	19.9	53.6	56.5	25.6	130	2.59	3.15
	88	2.43	3.84	12.8	4.88	10.9	6.75	2.42	22.5	20.7	54.8	58.0	25.5	134	2.71	3.38
SEM^2^		0.128	0.159	0.40	0.215	0.52	0.307	0.105	0.84	0.57	1.53	1.76	0.75	3.5	0.141	0.131
Main effects																
Enzyme addition																
		2.41	3.65	11.9	4.83	10.3	6.67	2.34	21.8	20.8	54.9	60.0^a^	24.5	136^a^	2.60	3.27
		2.39	3.68	12.0	4.93	10.5	6.54	2.47	22.0	19.9	52.7	56.4^b^	25.2	129^b^	2.63	3.24
Conditioning temperature, (°C)																
	60	2.34	3.47	11.6	4.92	10.7	6.86	2.53^a^	22.5	19.9^b^	52.3^b^	58.0	24.5	130	2.47	3.13^b^
	74	2.31	3.77	11.8	4.73	9.75	6.27	2.42^a^^,^^b^	20.8	19.8^b^	52.9^b^	57.1	24.8	130	2.58	3.17^b^
	88	2.54	3.74	12.5	4.97	10.7	6.69	2.26^b^	22.4	21.4^a^	56.1^a^	59.5	25.2	137	2.81	3.47^a^
Probabilities, *P* ≤																
Enzyme addition		0.836	0.824	0.727	0.575	0.697	0.590	0.127	0.852	0.076	0.087	0.018	0.302	0.028	0.814	0.806
Conditioning temperature		0.178	0.135	0.093	0.500	0.113	0.163	0.050	0.082	0.015	0.046	0.405	0.709	0.095	0.065	0.032
Enzyme × Conditioning temperature		0.221	0.572	0.502	0.285	0.176	0.622	0.219	0.244	0.325	0.082	0.294	0.359	0.152	0.293	0.494

a,b Means in a column not sharing common letters are different (*P* < 0.05).

1 Each value represents the mean of 6 replicates (2 birds per replicate).

2 Pooled standard error of mean.

3 Small intestine = duodenum + jejunum + ileum.

Supplemental Carb and CT did not interact (*P* > 0.05) to influence the gizzard pH or jejunal digesta viscosity, however, the gizzard pH tended (*P* = 0.065) to increase with increasing CT. Jejunal digesta viscosity was significantly (*P* < 0.05) influenced by the CT, as the diet conditioned at 88°C resulted in 10.1% (0.32 cP) greater digesta viscosity compared to the diets conditioned at 60° and 74°C.

## DISCUSSION

According to the analyzed GS contents of the conditioned-only and conditioned-pelleted diets (Table 1), conditioning the diets at 88°C itself may have contributed more to the formation of GS, leaving less room for pelleting to further contribute to the starch gelatinization. Abdollahi et al. (2010a) reported that GS content of corn- and sorghum-based diets markedly increased in conditioned-pelleted diets compared to conditioned-only diets (15.5 vs*.* 9.9 g GS per 100 g total starch). They attributed the increased GS to frictional heat and mechanical shear generated during the pelleting process and hypothesized that starch gelatinization takes place to greater extent during the actual pelleting steps (Heffner and Pfost, 1973; Abdollahi et al., 2010a).

Increasing CT from 60° to 88°C enhanced the pellet durability by 4.2 percentage points. This finding agrees with the literature (Cutlip et al., 2008; Abdollahi et al., 2010a, 2011) that attributed improved pellet quality to higher GS content in response to increasing CT. Svihus et al. (2005) suggested that an increase in diet viscosity, due partly to starch gelatinization, may enhance the binding capacity of feed particles leading to improved pellet quality. Although not assessed in the current study, the positive impact of protein denaturation at higher CT on pellet binding ability has also been acknowledged (Thomas et al., 1998; Abdollahi et al., 2013). Therefore, it can be speculated that a combination of factors induced by high CT resulted in the higher pellet durability in diets conditioned at 88°C. The poor pellet quality observed in the current study (PDI, 62.2%–66.4%), despite pellet binder inclusion in all the diets, is due mainly to the short pellets (less than 3.0 mm in length) manufactured. A linear relationship (r = 0.89) between pellet length and durability has been reported (Wood, 1987). According to Löwe (2005), the most sensitive part of the pellet is the surface of the breaks resulting from cutting the pellets exiting the die holes. The number of these sensitive breaks depends on the pellet length. Short pellets yield a higher number per mass than longer pellets creating higher abrasion and fines (Löwe, 2005). Therefore, shorter pellets may have lower PDI than pellets of higher length.

Regardless of the CT, addition of Carb to barley-based diets in the present study increased the WG by 30 g/bird and improved F/G by 6.5 points. Inborr and Bedford (1994), evaluated the supplementation of β-glucanase (0.0, 1.0, and 10 g/kg) to a barley-based diet conditioned at 75°, 85°, or 95°C for either 30 s or 15 min and reported no interaction between enzyme, temperature and time for growth performance. These researchers, however, reported a linear improvement in WG and F/G with increasing enzyme addition. Samarasinghe et al. (2000) studied the activity of cellulase enzyme in a barley-corn-soybean meal diet conditioned at 60°, 75°, and 90°C and reported that in diets prepared without exogenous enzymes, CT of 90°C, compared to 60°C, impaired WG of broilers (d 7-21) by 2.6 g/bird, daily FI by 2.0 g/bird and F/G by 4.1 points. Although exogenous enzyme activity was reduced by 71% in diets conditioned at 90°C compared to 60°C, added enzyme improved WG of the birds fed diets conditioned at 90°C by 11.1%, but not in those fed the diets conditioned at 60° or 75°C. Similarly, Cowieson et al. (2005) showed that increasing CT from 80° to 90°C in wheat-based diets without supplemental xylanase reduced WG by 154 g per bird and increased F/G by 9.0 points (1.94 vs. 2.03) in broilers (1–42 d). When xylanase was added to diets, WG and F/G improved in the birds fed diets conditioned at 85° or 90°C, but not in those fed the diet conditioned at 80°C. The improvements in growth performance due to the enzyme addition at higher CT reported by Samarasinghe et al. (2000) and Cowieson et al. (2005) were not, however, observed at lower CT, indicating a greater enzyme efficacy at higher CT or perhaps the performance was already maximised in birds fed diets conditioned at lower CT, leaving no room for enzymes to act. In contrast, as indicated by the lack of interaction between the Carb and CT in the current study for WG and F/G, the exogenous enzyme had similar efficacy at each CT, despite the low recovery in diets conditioned at 88°C. Moreover, due to the lack of effect of Carb on jejunal digesta viscosity, it can be speculated that enzyme action of hydrolysing the cell wall matrix (Bedford and Schulze, 1998) and generation of prebiotic oligosaccharides (González-Ortiz et al., 2017) contributed to the improvements in WG and F/G by supplemental Carb.

Feeding pelleted diets enhances economics of meat chicken production mainly through increased FI (Abdollahi et al., 2018) and subsequent improvements in growth rate and feed efficiency. However, the benefits of pellet feeding on bird performance partly depends on the CT applied during the pelleting process (Abdollahi et al., 2010a,b, 2011). In the current study, compared to diets conditioned at 60°C, birds offered diets conditioned at 88°C tended to consume 29 g less feed, gained 85 g less weight and showed deterioration of F/G by 8.1 points during the 21-d experimental period, while no differences were observed when the CT increased from 60° to 74°C. These observations are in agreement with previous studies (Samarasinghe et al., 2000; Creswell and Bedford, 2006) reporting deteriorated growth performance in broilers fed diets conditioned at temperatures above 80°C. Consistent with the present findings, Inborr and Bedford (1994) reported no effect from increasing CT of a barley-based diet from 75° to 85°C. However, when CT increased from 85 to 95°C, both WG and F/G were poorer. Loar II et al. (2014) reported that increasing the CT from 74° to 85° and 96°C in a corn-soybean meal diet deteriorated F/G by 3.0 (1.96 vs. 1.99) and 8.0 (1.96 vs. 2.04) points, respectively. Raastad and Skrede (2003) reported similar BW and F/G in 21-day-old broilers fed corn-wheat-oat-based diets conditioned at 69° and 78°C, but 5.4% lower BW and impaired feed efficiency by 11.5 points in those fed diets conditioned at 86°C.

The negative effect of high CT on digesta viscosity is believed to be primarily responsible for the poorer performance of birds fed high-temperature conditioned diets (Cowieson et al., 2005; Abdollahi et al., 2019). Lending support to this theory, CT at 88°C tended to lower FI by 29 g/bird compared to CT at 60°C, due possibly to the slower feed passage associated with greater digesta viscosity (McNab and Smithard, 1992; Almirall et al., 1995) in birds fed the diets conditioned at 88°C. Moreover, F/G of the birds was impaired by 2.4 points per 0.1 cp increase in jejunal digesta viscosity in response to the increasing CT from 60 to 88°C. In contrast, Abdollahi et al. (2010a) reported no effect of CT on F/G of birds fed corn- and sorghum-based diets conditioned at 60°, 75°, and 90°C, suggesting that deterioration of feed efficiency from high CT is more severe in diets based on viscous grains than those based on non-viscous grains.

Evaluating the influence of CT in corn- and wheat-based diets, Abdollahi et al. (2010b) reported that the reduced WG and FI in response to increasing CT from 60° to 75°C in corn-based diets was restored in the birds fed diets conditioned at 90°C. This effect was not, however, reported for wheat-based diets, with WG of birds fed diets conditioned at 75° and 90°C were lower than those fed diets conditioned at 60°C. In another study, Abdollahi et al. (2010a) reported that increasing CT from 60° to 75°C in both corn- and sorghum-based diets reduced WG, but it was restored in birds fed diets conditioned at 90°C. These observations led to the hypothesis that WG and FI responses of broilers fed diets conditioned at different temperatures represent a balance between the negative effects of high CT on nutrient availability on one hand and the positive effects of high CT on pellet quality on the other. Accordingly, the positive effects of conditioning at 90°C on pellet quality in non-viscous corn- and sorghum-based diets reported by Abdollahi et al. (2010a), was greater than the negative effects on nutrient utilization. On the other hand, improvements in pellet quality gained at higher CT in diets based on viscous grains, such as wheat (Abdollahi et al., 2010b) and barley, were insufficient to overcome the adverse effects of high CT on nutrient utilization caused by increased digesta viscosity. Apparently, the higher pellet quality achieved at 88°C in the current study did not reverse the negative impacts of high CT on WG and F/G.

Regardless of the CT, supplemental Carb enhanced starch digestibility by 1.15%. The positive effect of the supplemental Carb on starch digestibility in broilers fed barley-based diets has been reported previously (Bergh et al., 1999; Ravindran et al., 2007; Perera et al., 2019a,b). The enhanced starch digestibility, and the lack of Carb effect on jejunal digesta viscosity, implies the action of Carb on hydrolysing the cell wall matrix (Hesselman and Åman, 1986; Bedford, 1996) to release encapsulated starch granules, leading to better interactions with digestive enzymes. Enzyme addition increased the AMEn by 32 kcal/kg in the current study, which is parallel to the enhanced digestibility of starch as the main energy yielding nutrient in poultry diets. Both the improvement of AMEn in response to exogenous enzymes and the correlation with starch digestibility is recognized in the literature (Ravindran et al., 2007; Perera et al., 2019a).

Amerah et al. (2011) and Abdollahi et al. (2013) suggested that high temperature treatment of diets containing viscous grains impairs the ability of birds to utilize the nutrients through both increased digesta viscosity and reduced activity of the exogenous enzymes. In the current study, when the CT increased to 88°C, digestibility of all nutrients except fat and calcium reduced. Despite the recognized sensitivity of fat digestibility to high digesta viscosity (Edney et al., 1989; Almirall et al., 1995), the CAID of fat was only numerically reduced (by 2.69%) in response to increasing CT from 60° to 88°C.

Digestibility of N in the current study was influenced by the CT, where diets conditioned at 88°C had 5.3% lower N digestibility compared to those conditioned at 60°C. Increasing the CT to a certain extent benefits the protein digestibility through inactivating enzyme inhibitors and denaturing proteins to expose sites for enzyme attack (Camire et al., 1990; Abdollahi et al., 2013). However, extreme CT can reduce N digestibility by degradation of heat-labile amino acids, especially cysteine, followed by lysine, arginine, threonine, and serine (Papadopoulos, 1989). Loar II et al. (2014) reported that methionine, isoleucine and proline digestibility reduced by 3 to 5% in response to increasing CT from 74 to 85 and 96°C.

The CAID of starch in birds offered the diet conditioned at 88°C was 1.45% lower than those fed diets conditioned at 60°C. Abdollahi et al. (2010b) reported that conditioning wheat-based diets at 90°C lowered starch digestibility compared to diets conditioned at 60° and 75°C, while starch digestibility in corn-based diets was not affected by increasing CT. In a follow up study, Abdollahi et al. (2011) reported that CAID of starch in pelleted wheat-based diets decreased from 0.977 in diets conditioned at 60°C to 0.940 and 0.913 in diets conditioned at 75° and 90°C, respectively. The type of grain seems to affect the response also. Starch gelatinization increases the susceptibility for amylolytic degradation due to loss of crystalline structure (Svihus et al., 2005). Upon gelatinization, the starch granules are opened allowing the entrance of enzymes into the granule structure (Cornejo-Ramírez et al., 2018). Nevertheless, a linear relationship between the extent of gelatinization due to processing and starch digestibility is not evident, and hence higher GS contents does not necessarily mean a higher starch digestibility (Svihus et al., 2005). Moreover, crystallization of GS upon cooling to room temperature, known as retrogradation, re-associates starch molecules separated during gelatinization. Retrogradation can decrease starch digestibility (Abdollahi et al., 2013) by forming resistant starch that hinder enzymatic hydrolysis. Greater resistant starch content in response to elevated CT (90°C) has been reported in corn-, sorghum-, and wheat-based broiler diets (Abdollahi et al., 2010a; Abdollahi et al., 2011). Although resistant starch was not measured in the current study, it can be speculated that conditioning barley-based diets at temperatures above 74°C might have induced formation of resistant starch, negatively influencing starch digestibility.

Studies on the effect of CT on mineral digestibility are scant. Abdollahi et al. (2020) reported 37% reduction in calcium digestibility of broilers (d 1-21) fed wheat-based diets, and no effect on CAID of P in response to increasing CT from 60° to 90°C. In the current study, however, CAID of P decreased by 17.6%, while CAID of calcium remained unaffected when CT was increased from 60° to 88°C. It is reasonable to speculate that the higher digesta viscosity in birds fed diets conditioned at 88°C was partly responsible for the 17.6% reduction in CAID of P compared to those fed the diets conditioned at 60°C.

Compared to diets conditioned at 60°C, conditioning at 88°C reduced the IDE and AMEn by 186 and 56 kcal/kg, respectively. Reports on the effect of CT on energy utilization in broilers are not consistent and seem to be confounded by grain type. Abdollahi et al. (2010a) reported a grain type × CT interaction for energy utilization, where increasing CT from 60 to 90°C decreased AME of sorghum-based diets but had no effect on AME of corn-based diets. In a study with corn- and wheat-diets, Abdollahi et al. (2010b) reported no effect of CT on AME of diets conditioned at 60°, 75°, or 90°C. In a follow up study (Abdollahi et al., 2011), however, increasing CT of pelleted wheat-based diets from 60° to 90°C reduced AME by 74 kcal/kg. In agreement with these studies, the negative impact of high CT on IDE and AMEn in the present study showed a direct link to CAID of starch and can be attributed to the formation of resistant starch.

Birds offered diets with supplemental Carb had 6.0 and 5.1% shorter ileum and small intestine, respectively, compared to those fed nonsupplemented diets. Reduction in jejunal length in response to enzyme supplementation has been observed previously (Wu et al., 2004; Perera et al., 2020b) and was attributed to an enzyme-induced improvement in nutrient digestibility that decreased the need for digestive and absorptive capacity (Perera et al., 2020b).

Compared to the diet conditioned at 60°C, conditioning at 88°C resulted in a 10.7% reduction in cecal weight. Ceca enlarge as a consequence of increased fermentable material in the diet (Svihus, 2014). As hypothesized by Svihus et al. (2013), it can be speculated that viscous digesta in birds offered diets conditioned at 88°C impeded the passage of fermentable material into the ceca resulting in a significant reduction in the relative ceca weight. Feeding diets conditioned at 88°C increased the relative length of duodenum and jejunum by 7.5 and 7.3%, respectively, compared to the diets conditioned at 60°C. In agreement, Abdollahi et al. (2010b) reported a 6.3% longer small intestine in birds fed diets conditioned at 75° and 90°C compared to 60°C. This can be considered as the natural response to reduced availability of nutrients in diets exposed to higher CT.

Application of high temperatures during the conditioning process can increase the viscosity of feed and intestinal digesta through increased starch gelatinization (Svihus et al., 2005), enhanced release of encapsulated NSP (Cowieson et al., 2005), increased solubilization of NSP (García et al., 2008), presence of greater molecular weights due to less depolymerization of carbohydrates (Abdollahi et al., 2013) or destruction of both endogenous and exogeneous enzymes (Inborr and Bedford, 1994; Silversides and Bedford, 1999; Samarasinghe et al., 2000). Digesta viscosity is dependent not only on NSP concentration, but also on its molecular weight. A diet with a low content of soluble NSP might result in high viscosity if the NSP is of a high molecular weight (Cowieson et al., 2005). Impaired activity of both endogenous and supplemental enzymes due to high CT can reduce the extent of NSP depolymerization and contribute to an increase in molecular weight of NSP and consequently greater digesta viscosity (Silversides and Bedford, 1999; Cowieson et al., 2005). Therefore, it can be speculated that a combination of factors induced by high CT resulted in the 10.1% greater digesta viscosity in birds fed diets conditioned at 88°C compared to those fed diets conditioned at 60 and 74°C.

The proven impact of NSP-degrading enzymes in alleviating the higher digesta viscosity caused by extreme heat treatments of the wheat- (Silversides and Bedford, 1999; Cowieson et al., 2005) and barley- (Samarasinghe et al., 2000; Gracia et al., 2003; García et al., 2008) based diets was not observed in the current study. Samarasinghe et al. (2000) reported greater dietary viscosity in a barley-corn-soybean meal diet due to conditioning at 75° and 90°C compared to 60°C. Enzyme addition reduced the viscosity by 11, 14, and 17% in diets conditioned at 60°, 75°, and 90°C, respectively, showing greater magnitudes of response at high CT diets. Despite the lack of enzyme effect on digesta viscosity in the current study, WG, F/G, AMEn, and CAID of starch improved by supplemental Carb, suggesting the involvement of mechanisms other than reduction of digesta viscosity.

The thermostable enzymes used in this experiment have been used in previous studies in our laboratory (Perera et al., 2019a,b; 2020a,b) and were found to have high enzyme recoveries under high-temperature thermal processing. In contrast, extremely low enzyme recoveries were determined in diets conditioned at 88°C in the present study. It is difficult to provide a reason for this unexpected finding. In this study, CT was continuously measured and maintained at desired temperatures of 60°, 74°, and 88°C by adjusting the steam flow rate. The amount of heat required to achieve a particular CT depends on the difference between the preconditioning diet temperature (i.e., ambient temperature) and the desired temperature in the conditioning chamber. Accordingly, if the gap between ambient temperature and conditioning temperature is low, the amount of heat required to achieve that CT will be lower than if a high CT is desired. The current experiment was conducted during early spring and the diets were processed during a day with an average ambient temperature of <10°C. To achieve CT of 88°C, therefore, more heat (and moisture as heat was provided as steam) was applied (dos Santos et al., 2020) to the diet. This may explain, at least in part, the atypically low enzyme recoveries observed. Moreover, it may be that the amount of heat which is applied to the diet to achieve a certain CT is more important than final CT. Accordingly, evaluation of the recovery and stability of heat sensitive feed component such as enzymes in the future must be based on the amount of the heat applied to the feed *per se* rather than just a temperature reading. Moreover, sampling and experimental errors may also have contributed to the observed low activity of the enzymes. Despite the low enzyme activity in diets conditioned at 88°C, improved WG, F/G, starch digestibility and AMEn in response to Carb may suggest the potential contribution of contaminant activities present in the enzyme product that are neither listed nor assayed (Bedford, 2018).

## CONCLUSIONS

In conclusion, the efficacy of the test enzyme was similar at each CT as indicated by the lack of significant interactions between supplemental Carb and CT. Supplementation of Carb in barley-based diets improved WG, F/G, starch digestibility and AMEn in broiler starters. Steam-conditioning at 88°C negatively influenced the WG, F/G, ileal digestibility of N, starch, P, IDE and AMEn. Even though conditioning barley-based diets at 88°C delivered more durable pellets, nutrient utilization was seriously compromised, most likely due to the increased digesta viscosity, causing a substantial negative impact on growth rate and feed efficiency of the birds. Taken together with previous published data, it is evident that the response of viscous grains to increasing CT differs from those of non-viscous grains, highlighting the need to determine grain-specific optimum CT.

## DISCLOSURES

The authors declare that there is no conflict of interest.
